# Supplementary material for: An Improvement in Diagnostic Blood Culture Conditions Allows for the Rapid Detection and Isolation of the Slow Growing Pathogen Yersinia pestis
Source: Pathogens. 2022 Feb 16;11(2):255. doi: 10.3390/pathogens11020255 (PMC8874391; doi:10.3390/pathogens11020255)
Supplement: Supplementary file 1 [file pathogens-11-00255-s001.zip › pathogens-1592816-supplementary.pdf]

**Table S1. BACTEC Blood culture ingredients**

| | BD BACTEC™ Plus Aerobic/F | BD BACTEC™ Standard/10 Aerobic/F | BACTEC Standard Anaerobic/F | BACTEC Lytic/10 Anaerobic/F |
|---|---|---|---|---|
| **Processed Water** | 30 ml | 40 ml | 40 ml | 40 ml |
| **Soybean-Casein Digest Broth** | 3% | 3% | 3% | 2.75% |
| **Yeast Extract** | 0.25% | 0.3% | 0.4% | 0.2% |
| **Animal tissue digest** | | 0.01% | 0.01% | 0.05% |
| **Amino Acids** | 0.05% | | | |
| **Sugar** | 0.2% | Sucrose 0.1% | Dextrose 0.25% | Dextrose 0.2% |
| **Menadione** | | 0.00005% | 0.00005% | 0.00005% |
| **Hemin** | | 0.0005% | 0.0005% | 0.0005% |
| **Sodium Polyanetholsulfonate (SPS)** | 0.05% | 0.035% | 0.025% | 0.035% |
| **Vitamins** | 0.025% | vitamin B6 0.001% | | |
| **Antioxidants/Reductants** | 0.005% | | | |
| **Nonionic Adsorbing Resin** | 13.4% | | | |
| **Cationic Exchange Resin** | 0.9% | | | |
| **Sodium Bicarbonate** | | 0.04% | 0.04% | 0.04% |
| **Thiols** | | | 0.1% | 0.1% |
| **Sodium Citrate** | | | | 0.02% |
| **Sodium Pyruvate** | | | | 0.01% |
| **Saponin** | | | | 0.26% |
| **Antifoaming Agent** | | | | 0.035% |
